# Associations between *UGT1A1* and *SLCO1B1* polymorphisms and susceptibility to neonatal hyperbilirubinemia in Thai population

**DOI:** 10.1186/s12887-022-03311-4

**Published:** 2022-05-02

**Authors:** Chalirmporn Atasilp, Janjira Kanjanapipak, Jaratdao Vichayaprasertkul, Pimonpan Jinda, Rawiporn Tiyasirichokchai, Pornpen Srisawasdi, Chatchay Prempunpong, Monpat Chamnanphon, Apichaya Puangpetch, Natchaya Vanwong, Suwit Klongthalay, Thawinee Jantararoungtong, Chonlaphat Sukasem

**Affiliations:** 1 Chulabhorn International College of Medicine, Thammasat University, Pathum Thani, Thailand; 2 Division of Clinical Chemistry, Department of Pathology, Faculty of Medicine Ramathibodi Hospital, Mahidol University, Bangkok, Thailand; 3 Division of Pharmacogenomics and Personalized Medicine, Department of Pathology, Faculty of Medicine, Ramathibodi Hospital, Mahidol University, Bangkok, 10400 Thailand; 4 Laboratory for Pharmacogenomics, Clinical Pathology, Somdetch Phra Debharatana Medical Centre, Ramathibodi Hospital, Bangkok, Thailand; 5 Department of Pathology, Faculty of Medicine, Srinakharinwirot University, Nakhon Nayok, Thailand; 6 Division of Neonatology, Department of Pediatrics, Faculty of Medicine, Ramathibodi Hospital, Bangkok, Thailand; 7 Department of Clinical Chemistry, Faculty of Allied Health Sciences, Chulalongkorn University, Bangkok, Thailand; 8 Faculty of Medical Technology, Rangsit University, Pathum Thani, Thailand

**Keywords:** Genetic polymorphisms, UGT1A1, SLCO1B1, Hyperbilirubinemia, Neonates

## Abstract

Hyperbilirubinemia is the main mechanism that causes neonatal jaundice, and genetics is one of the risk factors of hyperbilirubinemia. Therefore, this study aims to explore the correlation between two genes, *UGT1A1* and *SLCO1B1*, and hyperbilirubinemia in Thai neonates. One hundred thirty seven neonates were recruited from Division of Clinical Chemistry, Ramathibodi Hospital. UGT1A1*28 and *6 were determined by pyrosequencing whereas, *SLCO1B1* 388A > G and 521 T > C genetic variants were determined by TaqMan® real-time polymerase chain reaction. Neonates carrying with homozygous (AA) and heterozygous (GA) variants in UGT1A1*6 were significantly related to hyperbilirubinemia development compared with wild type (GG; *P* < 0.001). To the combined of *UGT1A1*, total bilirubin levels in homozygous variant were higher significantly than heterozygous variant and wild type (*P* = 0.002, *P* = 0.003, respectively). Moreover, *SLCO1B1* combination was significant differences between the hyperbilirubinemia and the control group (*P* = 0.041). *SLCO1B1* 521 T > C variant provide protection for Thai neonatal hyperbilirubinemia (*P* = 0.041). There are no significant differences in UGT1A1*28 and *SLCO1B1 388A > G* for the different severity of hyperbilirubinemia. The combined UGT1A1*28 and *6 polymorphism is a strong risk factor for the development of severe hyperbilirubinemia in Thai neonates. Therefore, we suggest neonates with this gene should be closely observed to avoid higher severities of bilirubin.

## Introduction

Neonatal hyperbilirubinemia, one of the most common clinical problems in newborns, occurs up to 60% of healthy full-term newborns [1]. In general, elevated levels of total serum bilirubin can develop into severe neonatal jaundice that result in bilirubin-induced neurological damage such as hearing loss, athetosis, and rarely, intellectual deficits. Ultimately, severe cases may lead to seizures, coma, and death. The demographic, environmental, and genetic factors could account for risk of developing neonatal hyperbilirubinemia [2, 3].

The causes of neonatal jaundice can be due to the elevation of bilirubin, which is caused by the increased production or inability to metabolize and excrete it. This can be due to the immature liver of the newborn. Another cause is due to the decreased bilirubin uptake and conjugation due to the deficiency of the process of serum albumin binding to bilirubin, and carrying it to the liver [4]. However, there is a transient deficiency in newborns of the enzyme UDP-glucuronosyltransferase 1A1 (UGT1A1), which in turns leads to a reduced amount of ligandin, a bilirubin binding protein. Lastly, it may be caused by increased enterohepatic circulation. As conjugated bilirubin is excreted through the bile into the intestine, it is then deconjugated by a mucosal enzyme, β-glucuronidase, and reabsorbed into the enterohepatic circulation, before excretion via the stool. Newborns have slow intestinal motility, due to less gut flora, so this can be a cause of concern for major problems regarding excretion. Other causes include ABO incompatibility, hemolytic anemia and infection [5, 6].

The metabolism of bilirubin plays a huge role in hyperbilirubinemia. Firstly, when the individual component, “haem” is broken down into iron and biliverdin. Biliverdin is then reduced to create unconjugated bilirubin. As it is in the bloodstream, unconjugated bilirubin binds to albumin, facilitating the transport to the liver, and glucuronic acid is added by the enzyme UDP-glucuronosyltransferases [5, 7]. The first being UDP Glucuronosyltransferase Family 1 Member A1 (*UGT1A1*) which provides instructions for making enzymes called UDP-glucuronosyltransferases. This form conjugated bilirubin, which is soluble, and in turn can be excreted in the duodenum. Once inside, normal gut flora deconjugate bilirubin and convert it into urobilinogen. It is mostly oxidized by intestinal bacteria, and converted to stercobilin, which is then excreted through stool. The rest is then reabsorbed into the bloodstream as a part of the enterohepatic circulation [8].

The main factors that can be attributed to increased bilirubin may include race, acquired defects, and genetic polymorphism. Previous studies have been limited to only one genetic polymorphism [9, 10]. Therefore, two important genes, *UGT1A1* and *SLCO1B1* will be analyzed in this study**.** *UGT1A1* helps with the conjugation of bilirubin. Two single nucleotide polymorphisms of this gene will be studied. The frequency allele UGT1A1*28 is distinguished by the insertion of a TA in the TATAA box of the gene, consequently decreasing gene transcription [11]. There have been studies that shows the relationship between high bilirubin levels and UGT1A1*28 [12]. Another variant is UGT1A1*6, 211G > A at exon 1, has been reported as a risk factor for neonatal hyperbilirubinemia in Asians. Overall, these two single nucleotide polymorphism (SNP) have shown correlation in previous studies before [13].

Another gene, Solute Carrier Organic Anion Transporter Family Member 1B1 (*SLCO1B1*) provides instructions for making the protein “OATP1B1”, which transports compounds from the blood into the liver, so that they can be cleared from the body. Regarding bilirubin, *SLCO1B1* mediates the uptake of bilirubin, where it is conjugated and excreted from the body. Deficiencies in this gene can cause hyperbilirubinemia [14]. This includes the two SNPs, 388A > G and 521 T > C [15, 16].

Specifically, being able to identify the risk factors for neonatal jaundice can be crucial in developing treatment for this condition and minimize major consequences that may follow. The previous studies have been limited to only one type of gene in neonates, and there are only a few exploring the Thai population. This study aims to explore the correlation of the genetic variant of the two genes, *UGT1A1* and *SLCO1B1* causing hyperbilirubinemia in Thai newborns.

## Methods

### Patients

The subjects of case-control study were obtained between November 2019 and November 2020 at Division of Clinical Chemistry, Department of Pathology, Faculty of Medicine Ramathibodi Hospital, Mahidol University, Bangkok, Thailand. Eligible subjects including Thai neonates (≥37 weeks of gestation) were enrolled in this study. Exclusion criteria were causes of hyperbilirubinemia, such as hemolytic anemia, liver dysfunction, cholestasis, ABO and Rh incompatibilities, positive coombs test, glucose-6-phosphate dehydrogenase (G-6-PD) deficiency, hypothyroidism, cephalhematoma, encephalopathy and presence of neurological disorders in the brain.

Neonatal hyperbilirubinemia was defined as total serum bilirubin concentration of > 15 mg/dL beyond 14 days of life. The control group consisted of neonates who did not show prolonged hyperbilirubinemia beyond 14 days of life. The criteria was modified from the guideline of 2004 American Academy of Pediatrics [17].

This study was reviewed and approved by the Ethics Review Committee on Human Research of the Faculty of Medicine Ramathibodi Hospital, Mahidol University, Thailand (MURA2020/1514) and conducted in accordance with the Declaration of Helsinki.

### Molecular analysis

All leftover samples from total serum bilirubin determination were analyzed genetic polymorphisms. DNA extraction from clot blood samples was conducted using the Genomic DNA Mini Kit (Geneaid®, Geneaid Biotech Ltd., Taipei, Taiwan). Genomic DNA was quantified using NanoDrop ND-1000 Spectrophotometer (Thermo Fisher Scientific, DE, USA). The two single nucleotide polymorphisms (SNPs) at nucleotide 388A > G (rs2306283; on reference sequence NM_006446.4, assay ID: C:_1901697_20) and 521 T > C (rs4149056; on reference sequence NM_006446.4, assay ID: C:30633906_10) of *SLCO1B1* gene were determined by TaqMan® real-time polymerase chain reaction (RT-PCR) ViiA7™ system (Applied Biosystems, Life Technologies, Carlsbad, CA, USA) according to the manufacturer’s instructions.

The pyrosequencing (Qiagen, Japan) method was applied to detect the known variant sites in the *UGT1A1* gene: promoter area (UGT1A1*28) and nucleotide 211 (UGT1A1*6), protocol according to previously described method [18].

### Statistical analysis

Hardy–Weinberg equilibrium was assessed using Fisher’s exact and chi-square test for *UGT1A1* and *SLCO1B1* variants. Allele and genotype frequencies were determined by direct counting. Comparisons between the case group and the control group were performed with chi-square test. Mann–Whitney U test was performed according to difference of case-control groups and nonparametric data [Birth weight (g), Gestational age (week)]. One-way ANOVA was performed according to genetic groups and total bilirubin levels (mg/dl). All statistical analyses were performed by using SPSS version 21.0 (SPSS, Chicago, IL, USA). A *P*-value < 0.05 was considered to be statistically significant.

### Statement of confirmation

All methods aforementioned above were carried out in accordance with relevant guidelines and regulations.

## Results

### Clinical analysis

A total of 137 neonates were enrolled into the study. Sixty-seven neonates were classified into the hyperbilirubinemia group and 70 neonates were control group. Table 1 summarizes the demographic and clinical data between the hyperbilirubinemia group and control group. The factors listed here were gender, birth weight, gestational age, total bilirubin, and nutrition. The median of birth weight and gestational age were 3015.0 ± 770.0 g and 39.0 ± 1.0 weeks, respectively for case group and 2995.0 ± 695.0 g and 38.0 ± 3.0 weeks, respectively for control group. The average total bilirubin of the case group was 18.8 ± 2.6 mg/dL higher than the control group (10.7 ± 3.5 mg/dL). In the category of nutrition, there was statistically significant values between the hyperbilirubinemia and control group (*P* = 0.013). However, there were no obvious differences between gender, birth weight, and gestational age, with a *P*-value of 0.763, 0.352 and 0.442, respectively.

**Table 1:** Demographic and clinical data (*N* = 137)

Factors	Hyperbilirubinemia group *n* = 67 (%)	Control group *n* = 70 (%)	*P-*value
Gender			
Male	35 (50.0)	35 (50.0)	0.763
Female	32 (47.8)	35 (52.2)	
Birth weight (g)	3015.0 ± 770	2995 ± 695	0.352
Gestational age (week)	39.0 ± 1	38.0 ± 3	0.442
Total bilirubin (mg/dL)	18.8 ± 2.6	10.7 ± 3.5	< 0.001*
Nutrition			
Breast feeding	30 (42.3)	41 (57.7)	0.013*
Formula feeding	3 (100)	0 (0)	
Mixed feeding	34 (54.0)	29 (46.0)	

* *P*-value < 0.05 was considered to be statistically significant

### The genotype and allele frequency of *SLCO1B1* and *UGT1A1* variants

The analysis of the genotype and allele frequency of *SLCO1B1* and *UGT1A1* variants are shown in Table 2. The allele frequencies of *SLCO1B1* 388A > G, 521 T > C, UGT1A1*28 and *6 were 0.79, 0.13, 0.17, and 0.13, respectively. Genotyping of *SLCO1B1* 388A > G was firstly mentioned. Homozygous variant (GG) was the most abundant, showing 84 (61.3%) and followed by 48 (35.0%) in heterozygous variant (AG) and 5 (3.6%) in wild type (AA). For *SLCO1B1* 521 T > C, TT, TC, and CC were also measured, and the genotype frequencies were 105 (76.6%), 29 (21.2%) and 3 (2.2%), respectively. The next gene explored was UGT1A1*28. The genotypes of TA6/TA6, TA6/TA7, and TA7/TA7 were 90 (65.7%), 46 (33.6%) and 1 (0.7%), respectively. The genotyping of UGT1A1*6211G > A, was GG, GA, and AA, the frequencies are 107 (78.1%), 25 (18.2%) and 5 (3.6%) respectively.

**Table 2:** Genotype and allele frequency of *SLCO1B1* and *UGT1A1* variants

Genetic polymorphism	Allele frequency	Genotype frequency (%)
*SLCO1B1* 388A > G		
A allele	0.21	
G allele	0.79	
AA		5 (3.6)
AG		48 (35.0)
GG		84 (61.3)
SLCO1B1 521 T > C		
T allele	0.87	
C allele	0.13	
TT		105 (76.6)
TC		29 (21.2)
CC		3 (2.2)
Combined SLCO1B1^a^		
Normal function		105 (76.6)
Intermediate function		29 (21.2)
Low function		3 (2.2)
UGT1A1*28		
TA6 allele	0.83	
TA7 allele	0.17	
TA6/TA6		90 (65.7)
TA6/TA7		46 (33.6)
TA7/TA7		1 (0.7)
UGT1A1*6211G > A		
G allele	0.87	
A allele	0.13	
GG		107 (78.1)
GA		25 (18.2)
AA		5 (3.6)
Combined UGT1A1 ^b^		
Wild type		66 (48.2)
Heterozygous variant		59 (43.1)
Homozygous variant		12 (8.8)

^a^ Normal function consists of *1a/*1a, *1a/*1b, *1b/*1b; Intermediate function consists of *1a/*5, *1a/*15, *1b/*15; Low function consists of *5/*5, *5/*15, *15/*15

^b^ Combined UGT1A1 wild type (*1/*1); heterozygous variant (*1/*28, *1/*6); homozygous variant (*28/*28, *28/*6, *6/*6)

Regarding combined *SLCO1B1*, the frequency values for normal, intermediate and low function were as follows; 105 (76.6%), 29 (21.2%) and 3 (2.2%), respectively. Lastly, combined *UGT1A1* frequencies were measured. The wild type, heterozygous, and homozygous variant were genotyped to 66 (48.2%), 59 (43.1%) and 12 (8.8%) respectively.

### The correlation between case-control group and genetic factors

Table 3 was showed the distributions for genetic factors for neonatal hyperbilirubinemia. The *SLCO1B1* 521 T > C variant showed significantly a low risk of neonatal hyperbilirubinemia in neonates (*P* = 0.041). The combined of *SLCO1B1* was significantly related to severe hyperbilirubinemia (*P* = 0.041). In this study found that all neonates carrying homozygous variant in UGT1A1*6 had high development of hyperbilirubinemia (5/5; 100%; *P* < 0.001). Moreover, *UGT1A1* combination was significantly increases the risk of hyperbilirubinemia (*P* = 0.005).

**Table 3:** Correlation between case-control group and genetic factors

Factors	Hyperbilirubinemia group n = 67 (%)	Control group n = 70 (%)	*P-*value
SLCO1B1 388A > G			
AA	1 (20.0)	4 (80.0)	0.173
AG	21 (43.8)	27 (56.3)	
GG	45 (53.6)	39 (46.4)	
SLCO1B1 521 T > C			
TT	57 (54.3)	48 (45.7)	0.041*
TC	9 (31.0)	20 (69.0)	
CC	1 (33.3)	2 (66.7)	
Combined SLCO1B1			
Normal function	57 (54.3)	48 (45.7)	0.041*
Intermediate function	9 (31.0)	20 (69.0)	
Low function	1 (33.3)	2 (66.7)	
UGT1A1*28			
TA6/TA6	49 (54.4)	41 (45.6)	0.097
TA6/TA7	18 (39.1)	28 (60.9)	
TA7/TA7	0 (0)	1 (100)	
UGT1A1*6211G > A			
GG	44 (41.1)	63 (58.9)	< 0.001*
GA	18 (72)	7 (28)	
AA	5 (100)	0 (0)	
Combined UGT1A1			
Wild type	31 (47.0)	35 (53.0)	0.005*
Heterozygous variant	26 (44.1)	33 (55.9)	
Homozygous variant	10 (83.3)	2 (16.7)	

^a^ Normal function consists of *1a/*1a, *1a/*1b, *1b/*1b; Intermediate function consists of *1a/*5, *1a/*15, *1b/*15; Low function consists of *5/*5, *5/*15, *15/*15

^b^ Combined UGT1A1 wild type (*1/*1); heterozygous variant (*1/*28, *1/*6); homozygous variant (*28/*28, *28/*6, *6/*6)

* *P*-value < 0.05 was considered to be statistically significant

**Fig. 1:**
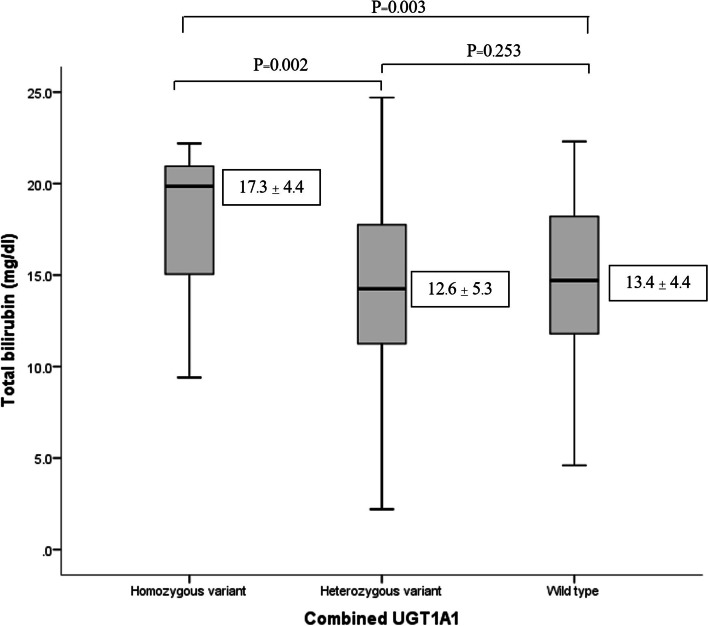
Correlation between combined UGT1A1*28 and *6 and total bilirubin levels in Thai neonates; Combined *UGT1A1* wild type (*1/*1); heterozygous variant (*1/*28, *1/*6); homozygous variant (*28/*28, *28/*6, *6/*6)

**Fig. 2:**
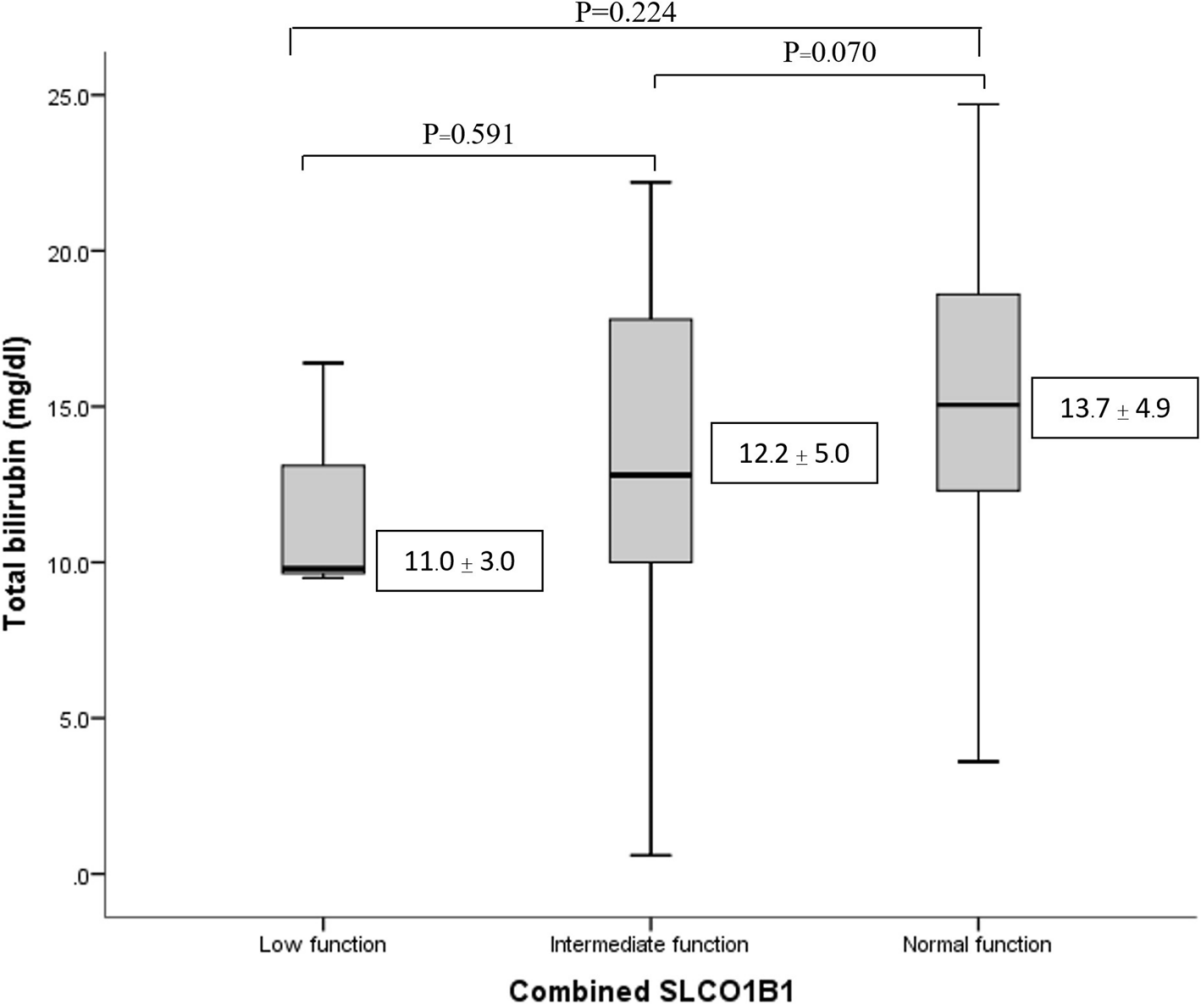
Correlation between combined *SLCO1B1* and total bilirubin levels in Thai neonates; normal function consists of *1a/*1a, *1a/*1b, *1b/*1b; intermediate function consists of *1a/*5, *1a/*15, *1b/*15; low function consists of *5/*5, *5/*15, *15/*15

Similar to Fig. 1, this box plot diagram shows that neonate carrying homozygous variant of combined *UGT1A1* had a significantly increased of total bilirubin levels when compared with heterozygous variant and wild type (*P* = 0.002, and 0.003, respectively). As shown in Fig. 2, this box plot diagram shows the results of combined *SLCO1B1* with low (5/*5, *5/*15, *15/*15), intermediate (*1a/*5, *1a/*15, *1b/*15,), and normal (*1a/*1a, *1a/*1b, *1b/*1b) function and the total bilirubin (mg/dL) measured. There was no significant association between combined *SLCO1B1* and total bilirubin levels. However, our results shown that neonates with low function had a decreasing trend in total bilirubin levels compared with intermediate and normal function. The average of total bilirubin in low, intermediate and normal function as follow: 11.0 ± 3.0 mg/dL, 12.2 ± 5.0 mg/dL, and 13.7 ± 4.9 mg/dL, respectively.

## Discussion

In this study, the correlation between hyperbilirubinemia and two genes, *SLCO1B1* and *UGT1A1* variants were investigated in Thai neonates. Our results showed that combined UGT1A1*28 and *6 is a high-risk factor for developing neonatal hyperbilirubinemia. We were the first study to conduct on combined *UGT1A1* variants and its effects on Thai neonatal hyperbilirubinemia. Regarding *SLCO1B1*, a trend was evident, as the total bilirubin levels were a decreasing in low function compared with intermediate and normal function.

UGT1A1*28 (A(TA)7TAA) is a variant allele that is commonly found in African-Americans (0.42–0.45 allele frequency), and less in Asian populations (0.09–0.16 allele frequency) [19, 20]. It provides instructions for making UDP-glucuronosyltransferase, which is crucial in the overall process of converting unconjugated bilirubin. However, our results showed that UGT1A1*28 is not a risk factor for developing neonatal hyperbilirubinemia. Similarly, to a meta-analysis by Li H, et al. [21], concluding that the gene polymorphism of UGT1A1*28 might not be associated with the risk of neonatal hyperbilirubinemia.

UGT1A1*6211G > A, another SNP variant, was explored. The results from Prachukthum S, et al. [22], reported that UGT1A1*6 was an important risk factor for developing jaundice in infants. It found that infants who were carrying homozygous (AA) and heterozygous (GA) variants were more susceptible to develop hyperbilirubinemia when compared with wildtype (GG). Yanagi T, et al. [23], showed that UGT1A1*6 is a risk factor for prolonged unconjugated hyperbilirubinemia in Japanese preterm infants. Moreover, Nguyen TT, et al. [13], revealed that bilirubin levels in the patient carrying homozygous c.211G > A was significantly higher that heterozygous variant and wild type. Our results demonstrated that all Thai neonates carrying the homozygous variant in UGT1A1*6 were significantly classified into the hyperbilirubinemia group.

For our results regarding combined UGT1A1*28 and *6, it showed that these two genes were strongly significantly associated with hyperbilirubinemia (*P* = 0.005). The total bilirubin levels in the homozygous variant were significantly higher compared with heterozygous variant and wildtype.

The second gene studied was *SLCO1B1*. This gene provides instructions for making a protein, OATP1B1, an influx transporter responsible for the transportation of compounds in the bloodstream to the liver [24]. The allele frequency of *SLCO1B1* 388 A > G in this study was 0.79, similarly to the Han Chinese population having an allele frequency of 0.64 reported by Liu et al. [11]. Moreover, Bai J, et al. [25], found that the 388 G > A variant of the *SLCO1B1* gene was associated with infant hyperbilirubinemia in Chinese. However, the data from our study indicates that there were no statistically significant differences in risk factor of neonatal hyperbilirubinemia and *SLCO1B1* 388 A > G variant. Similar to Amandito R, et al. [26], demonstrated that there was no statistically significant differences between occurrence of *SLCO1B1* 388 A > G and hyperbilirubinemia in newborns. In *SLCO1B1* 521 T > C variant, there was a significant correlation between hyperbilirubinemia and *SLCO1B1* 521 T > C (*P* = 0.041). Similarity to a systematic review with meta-analysis of Liu J et al. [27], reported that the *SLCO1B1* 521 T > C variant protective factor against hyperbilirubinemia in Chinese neonates.

Regarding combined *SLCO1B1*, there was a decreasing trend of total bilirubin levels in normal (*1a/*1a, *1a/*1b, *1b/*1b), intermediate (*1a/*5, *1a/*15, *1b/*15), low (5/*5, *5/*15, *15/*15) function respectively. The relationship between low function neonates, having lower total bilirubin levels than that of intermediate and normal function, was evident.

In the present study, total bilirubin levels were significantly between case and control groups. In case group, mean of total bilirubin levels were 18.8 ± 2.6 mg/dL, which had higher total bilirubin levels than control group (10.7 ± 3.5 mg/dL). The total bilirubin levels in control group showed slightly high levels in Thai neonates. There was a nutrition significance to be noted in this study. The results showed that there was a correlation between nutrition and neonatal hyperbilirubinemia in Thai neonates (*P* = 0.013). The finding was consistent with Bratton S et al. [28], stating that breast milk may cause jaundice in newborns in their first week of life. There is limited research regarding formula and mixed-feeding and its association with neonatal jaundice.

In addition, some limitations of this study were regarding small sample size, which does not represent the whole population. Large sample size could be studied in further study. Our study was also limited to only two genes, *SLCO1B1* and *UGT1A1*, so other genes related with hyperbilirubinemia could be investigated in further studies. Since this was a retrospective study, some clinical data were also missed, including nutrition.

## Conclusion

The combined UGT1A1*28 and *6 polymorphism was a strong risk factor for hyperbilirubinemia in Thai neonates. Therefore, we suggest neonates with this gene should be closely observed to avoid higher severities of bilirubin.

## Data Availability

Full data set and other materials on this study can be obtained from the corresponding author on reasonable request.
